# Type of Diet and Sports Supplements in Padel Players According to Level of Competition and Sex

**DOI:** 10.3390/nu15163633

**Published:** 2023-08-18

**Authors:** Víctor Toro-Román, Alejandro Muñoz, Antonio Zoido, Bernardino J. Sánchez-Alcaraz, Francisco Grijota, Diego Muñoz

**Affiliations:** 1 Faculty of Sports Sciences, University of Extremadura, 10003 Caceres, Spain; vtoro@unex.es (V.T.-R.); diegomun@unex.es (D.M.); 2 Exercise Physiology Group, Exercise and Sport Sciences, Faculty of Health Sciences, Universidad Francisco de Vitoria, 28223 Madrid, Spain; alejandro.munoz@ufv.es; 3 Az-Nutrición, Az Clinic, 29015 Málaga, Spain; info@aznutricion.com; 4 Faculty of Sports Sciences, University of Murcia, 30100 Murcia, Spain; bjavier.sanchez@um.es

**Keywords:** racquet sports, micronutrients, performance, nutrition

## Abstract

Padel is the world’s fastest growing racket sport. However, the analysis of the intake of sports supplements in padel players is scarce. The aim of this research was to analyse and compare the type of diet and the use of sports supplements in padel players according to their competition level and sex. A total of 123 players (94 men and 29 women) participated in the study. Subjects were divided according to their sex and competition level. All participants completed an anonymous questionnaire on diet type and nutritional supplement intake. There were differences found in diet type between competition levels. Regarding players’ sex, differences in the number of supplements consumed were reported (*p* < 0.01). Relationships were found between the number of supplements ingested and the perceived effectiveness of supplements with frequency and time of training (*p* < 0.05). Creatine in men (≈15%) and vitamin complexes (≈10%) in women were the most used supplements. Lower level padel players do not adapt their diet to the physical demands of padel. Male padel players use a greater number of supplements than female padel players. It is important that nutrition specialists advise players to control diets and supplement.

## 1. Introduction

Padel is an intermittent racquet sport that is played by pairs on an artificial turf (20 × 10 m) surrounded by glass walls and metal mesh on which the balls bounce [1]. The popularity and the number of padel licenses have grown exponentially over the last few years [2,3], as has the amount of research related to the sport [4,5].

During a padel match, short actions of great intensity (0.7–1.5 s) are combined with long and less intense actions (9–15 s), alternating with pauses and breaks between points that normally last 10–20 s [6]. Padel is characterised by being a predominantly aerobic sport, which includes short play actions of high and very high intensity, in which the phosphagen system prevails [7,8]. In fact, low intensity and rest periods occupy about 60% of the playing time, with an effort/rest ratio of 1:2 1:2.5 [9]. These game characteristics provoke acute responses within the organism. Firstly, with regard to cardiorespiratory parameters, oxygen consumption during a match amounts to around 40–50% of the maximum oxygen consumption [7,8]. As for heart rate (HR), an average HR of 151 ± 8 bpm (76% of the maximum heart rate) has been recorded [10]. Lactate concentration appears to follow a similar stable pattern during a padel match (start: 1.83–1.90 mmol/L; end: 2.40–3.38 mmol/L) [4,7]. From a biochemical point of view, previous studies have observed significant increases of glucose, urea, creatinine, creatinine kinase (CK) and uric acid [10] levels after padel matches. However, concentrations of electrolytes such as sodium (Na), potassium (K), chlorine (Cl) and magnesium (Mg) decreased after padel matches [10]. Finally, regarding physical parameters, it seems that professional players have lower levels in the vertical jump with counter-movement after playing a padel match [11]. These data confirm that players’ physical performance and recovery are compromised during and after a padel game. Therefore, it is important to implement strategies to improve players’ recovery and performance, especially during tournaments.

The athlete’s nutritional intake has a direct influence on increasing adaptations to training, improving performance and recovery [12]. Nutrition is considered a fundamental factor in the overall development of an athlete [13]. It is important to understand the eating habits of athletes, as they influence energy consumption, nutrient intake and hydration status [12,14]. Meanwhile, sports supplements or nutritional ergogenic aid aims to improve sports performance without exerting harmful effects on the individual [15]. The intake of sports supplements is more common in elite athletes, with the intake being similar in men and women [16].

Nutritional recommendations and habits have already previously been analysed in racquet sports [12,17]. According to Fleming et al., [12] on match days, carbohydrates were prioritised before a match. During the matches, all players adopted a nutritional strategy based on water (94%) and bananas (86%). Regarding sports supplements, sports drinks (80%) and energy gels (26%) rich in carbohydrates were the most frequently used during long games. In general, the most commonly used sports supplements in racquet sports are creatine and caffeine [18], as well as sodium bicarbonate [19]. However, specific information for padel is scarce. To date, only one study has evaluated the influence of a sports supplement (caffeine) on performance parameters in padel players [20]. Caffeine intake (6 mg/kg) produced a similar perceived exertion and hand grip strength to the control group. On the other hand, in the padel specific test, the results were slightly better in the caffeine intake group. In addition, only one study has analysed the use of dietary supplements in padel players [21]. This study concluded that players had a high prevalence of use of dietary supplements—isotonic and energy drinks being the most predominant. However, type of diet was not evaluated and differences between competition levels among players were not considered.

As athletes often use dietary supplements without a clear understanding of their effects [22], and bearing in mind the lack of studies regarding the type of diet and the use of sports supplements in padel, it seems important to provide information on dietary patterns and the use/purchase of dietary supplements in athletes. Analysis of diet type and sports supplement intake could help coaches and nutrition professionals to provide nutritional guidance to players, as well as educate and establish nutritional habits that improve performance and overall health. Thus, the aim of this study was to analyse and compare the type of diet and the use of sports supplements in padel players according to their competition level and sex.

## 2. Materials and Methods

### 2.1. Participants

A total of 123 padel players voluntarily participated in the present study. All participants were informed of the purpose of the study. The sample size is higher for a population of 95,000 players (based on the number of licences of the Spanish Padel Federation; Madrid, Spain) for a confidence interval of 95% and a margin of error of 10%. The study design was approved by the Biomedical Ethics Committee of the University of Extremadura (Caceres, Spain), following the guidelines of the Helsinki ethical statement, updated at the World Medical Assembly of Fortaleza, Brasil (2013), for research on human beings (154/2020). To ensure their anonymity, each participant was assigned a code for the collection and treatment of the samples. Table 1 shows the characteristics of the participants and Table 2 shows the characteristics of the weekly training carried out by the study subjects.

Inclusion criteria to participate in the study were as follows: (i) being over 18 years old; (ii) having at least one year of experience playing padel; (iii) not having any pathologies and (iv) not having any allergies or food intolerance that modifies the type of diet or prevents the intake of certain foods.

### 2.2. Study Design

A descriptive and cross-sectional study was carried out on the type of diets and habitual consumption of sports supplements. A questionnaire was distributed through social networks by professionals from the world of padel (researchers, institutions, players, coaches and nutritionists) informing the players of this study’s existence and inviting them to participate.

All players who participated in this research did so voluntarily, completing a questionnaire on the type of diet and the intake of sports supplements [23]. The players completed the questionnaire between February and March in a web format prepared to facilitate its distribution.

To divide the participants according to competition level, the league they played in was considered (low level: second/third regional division; middle level: first regional division; professional: participation in the World Padel Tour (WPT) circuit or similar).

### 2.3. Instrument

The questionnaire used in this study is based on a previous questionnaire [23]. That questionnaire has previously been used in other racquet sports [19], as well as individual and collective sports [24,25,26,27] and mountain runners [28]. It should be noted that the form obtained a 54% methodological quality score in a review by Knapik et al. [16] in which only 57 of the 164 different questionnaires they reviewed for the study of the consumption of sports supplements received their approval.

The questionnaire [28] contained a total of 24 questions divided into three main sections: The first included anthropometric data (e.g., height, age and weight) and personal data (e.g., sex) (4 questions); the second covered the practice of sport and its context (8 questions; e.g., years of practice, frequency of training, number of competitions, …); and the last and most extensive was related to the type of diet and consumption of sports supplements (12 questions). This part included, among other questions: diet type (low fat, ketogenic, hyperprotein, etc.), diet adaptation according to physical demands, sports supplements consumed, why they were consumed, who advised said consumption, where they were bought, when they were taken and the perception of results after consumption by the players.

### 2.4. Statistical Analysis

Quantitative data are expressed in mean ± standard deviation and qualitative data in frequencies (percentage). The statistical analysis was performed using the software SPSS v.20.0 (SPSS Inc., Chicago, IL, USA.). First, a normality analysis was carried out using the Kolmogorov Smirnov test to perform a comparative analysis between quantitative variables. Subsequently, to determine the differences, a two-way ANOVA (sex and level) and a one-way ANOVA (level) were used. The Bonferroni post hoc test was used. Meanwhile, the Chi-square test (χ^2^) was used to compare the differences between the qualitative variables. Finally, the Pearson correlation test (*r*) was used to analyse the correlations between the variables studied. The post hoc statistical power analysis used the GPower program (version 3.1). The statistical significance level was set to *p* < 0.05.

## 3. Results

Post hoc statistical power for an alpha set at *p* < 0.05 was: 0.24 for chi-square tests; 0.83 for two-way ANOVA tests; 0.81 for Pearsons’s correlation coefficients tests.

There were significant differences between levels of competition regarding special diets and diets adapted to sports demand (*p* < 0.01) (Table 3). There were no significant differences between sexes (Table 3).

There were significant differences between levels of competition in the use of sports supplements and days of consumption (*p* < 0.05) (Table 4). The intake of sports supplements increases depending on the levels of competition. Nevertheless, players of lower level usually ingest sports supplements daily. Regarding sex, there were differences in the number of sports supplements consumed, with men ingesting more supplements (between 2–3 supplements) (Table 4).

**Figure 1:**
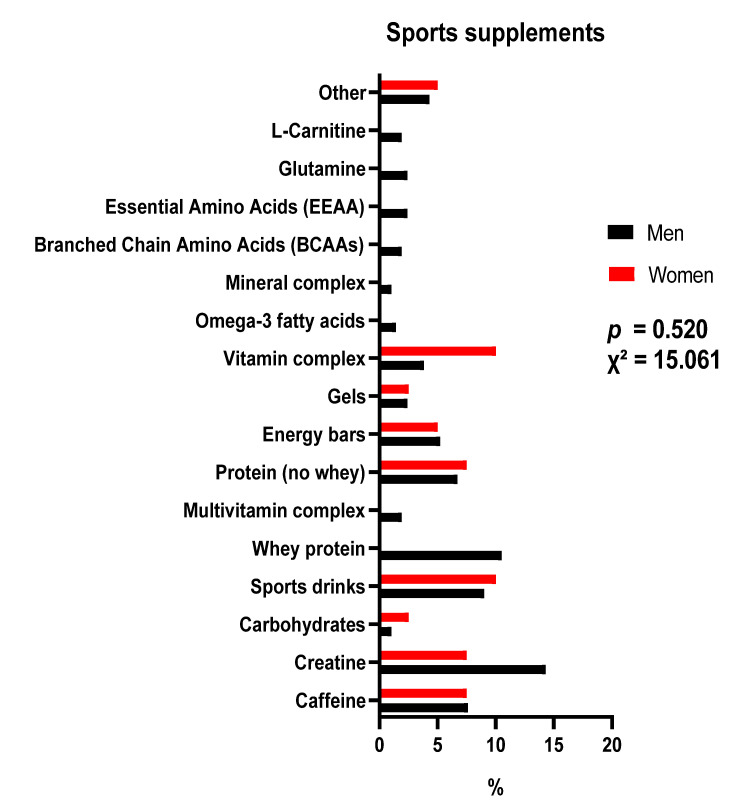
Sports supplements used according to sex, expressed in percentages. Red: female; black: male; others: nitrates, arginine, citrulline, vegetable protein and beta alanine.

**Figure 2:**
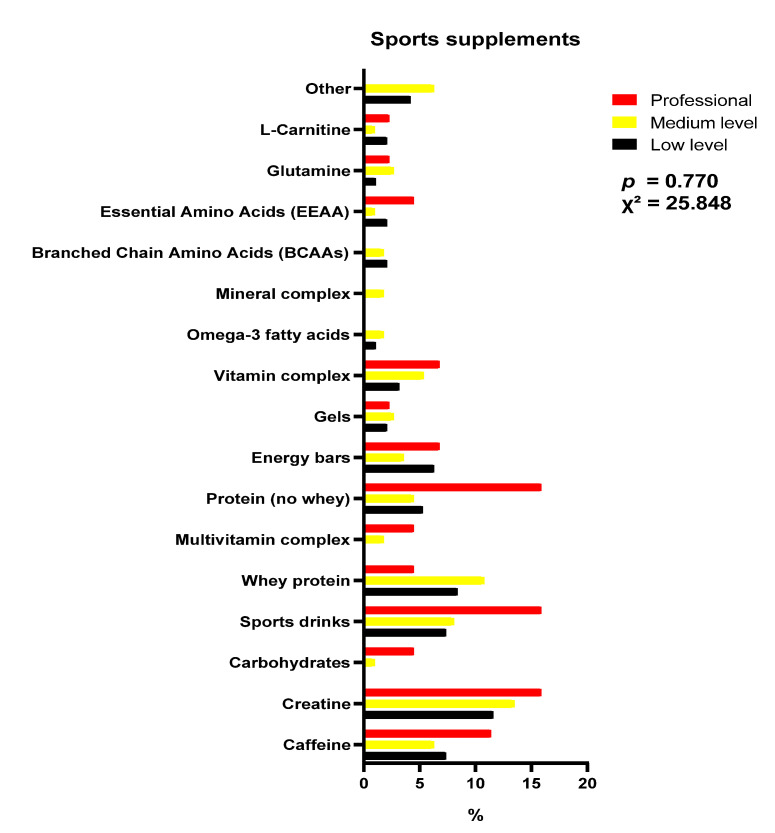
Sports supplements used according to competition level, expressed in percentages. Black: low level; yellow: medium level; red: professional level; others: nitrates, arginine, citrulline, vegetable protein and beta alanine.

Figure 1 show the type of sports supplement according to sex. Figure 2 shows the data on the type of sports supplements depending on the level of competition. There were no significant differences. Creatine in men and multivitamin complex in women were the most used. Regarding the level of competition, creatine was the most consumed supplement in all levels of competition.

The following shows information on acquisition, motivation and the person in charge of controlling sports supplements in the study participants (Table 5). There were no significant differences.

Below are the results regarding the perception of sports supplements’ effect on the participants and the number of supplements ingested (Table 6). There were significant differences between sex in the number of sports supplements consumed (*p* < 0.05).

Finally, below is the information on the correlations between the variables analysed according to sex (Table 7). There were significant positive correlations in men between supplements intake and age (*p* < 0.001), whereas in the case of female players, there were direct correlations between the number of supplements ingested and the total daily training time (*p* < 0.05), as well as between age and intake of sports supplements (*p* < 0.05). In addition, there is a positive correlation between the perception of supplement effectiveness and total training time (*p* < 0.05) (Table 7).

## 4. Discussion

The objectives of this research were (i) to analyse and compare the type of diet in padel players according to their level of competition and sex and (ii) to analyse and compare the use of sports supplements in padel players according to their level of competition and sex. Among the main findings were the following: (i) there were differences in diet type between levels of competition and sex; (ii) positive relationships were found between the number of supplements taken and the perceived effectiveness of supplements with frequency and time of training; and (iii) creatine in male and vitamin complexes in female padel players were the most commonly used supplements.

It is important to understand the eating habits of athletes as they could influence the intake of energy and nutrients and affect the body’s hydration status [12]. Several studies have researched eating habits and nutritional knowledge in different sports [29,30], including racquet sports [12,31]. However, to date, there exists little information regarding padel. The prevalence of the use of sports supplements has also been the subject of much research [32,33]. Overall, in Spain, the popularity of sports supplement intake ranges from approximately 48–81% [22], including protein and multivitamins. The reasons reported by athletes for using dietary supplements are diverse, although they are mainly related to health-related problems and improved physical and mental performance [22]. The risks of dehydration and potential performance consequences can be unique to track and field athletes, as their fluid needs are highly variable because of the diverse nature of events, training programs, and individual differences among athletes. A review of the effects of dehydration on muscle strength, power and high intensity anaerobic capacity showed that dehydration could impact on strength and power [34]. On the other hand, the evidence supports a variety of dietary strategies to improve sports performance [35]. Nutritional strategies to improve performance include optimizing the intake of macronutrients, micronutrients and fluids, including their composition and spacing throughout the day. The importance of individualized or personalized dietary counselling is increasingly recognized, with nutritional strategies that vary according to sport. In addition, sports supplements can improve performance, provided they are used properly [36]. In racquet sports, it has been reported that caffeine and creatine are the most studied supplements in the scientific field [18,37]. However, for padel, the information is scarce. Unlike previous studies in similar sports [18,19], this analysis has counted on many participants.

In the present study, many participants did not follow a special diet or adapted consumption to the specific demands of this sport. Ventura-Comes et al. [38] reported low intake of carbohydrate-rich foods below the recommended guidelines in elite Spanish squash players. The absence of a diet adapted to physical demands could be related to limited nutritional knowledge to adopt optimal dietary practices then. The nutritional knowledge of an athlete is one of the modifiable determinants of dietary behaviour [39]. The association between nutrition knowledge and dietary behaviour is multifaceted and is influenced by many other factors such as experience, financial status, friendships, sports culture, access to food and culinary skills [40]. The control and adaptation of dietary intake is critical to perform properly and speed up the recovery process. In relation to the above, among the participants who followed a special diet, in male players the hyperprotein and low-fat diets predominated, whereas in female players the Mediterranean diets high in low fat CHO (carbohydrates) prevailed. It is well known that a diet high in CHO increase in muscle glycogen stores, which contributes to optimal performance, particularly in endurance activities [41]. Similarly, a low-CHO diet can impair high-intensity exercise and endurance performance, which is critical for racquet sports. Previous studies conducted on tennis players reported other studies in tennis players where 51% opt for balanced meals (consisting of CHO, fats and proteins with some micro-nutrient considerations) whereas 27% choose foods high in CHO [12]. Regarding basketball and badminton players, adherence to a Mediterranean diet was higher than the average [42]. Much the same occurs among professional canoeists, who recorded moderate or excellent adherence to the Mediterranean diet [43]. Finally, an important aspect to consider is culture and religion. Athletes worldwide come from various sporting, religious and cultural backgrounds, so cultural beliefs, traditions and values can influence their food choices. For some athletes, family traditions and ethnicity are crucial aspects of food choice. Culture within sport can influence food choice where traditions and beliefs are strong, and the value of nutrition may not be recognized [44].

The proportion of consumption of sports supplementation differs depending on a multitude of variables, such as sports modality [16]. Although the results of our study place the consumption of supplements in a range of 50% for low and medium competition levels and 87% for professionals, in other sports modalities, the prevalence in the use of supplements was 53–56% in basketball players [45], 50–58% in football players [46] and 46% in volleyball players [45]. The prevalence in racquet sports such as tennis and squash was much higher (approximately 81%) [18,19]. This present study revealed that men’s average use of sports supplements was higher. In addition, the use of sports supplements increased as the level of competition increased. With regard to other sports, the average of sports supplements in sailors was 3.9 [47], in squash players 8.4 [19], and in bodybuilders, 20 [48]. Contrary to our study, in fencers the total intake of supplements did not differ according to sex or the level of competition [49]. However, other reviews did report sex differences in sports supplement intake [16,50]. Compared to male players, in the present study, female players used vitamin supplements in greater quantity. These results are similar to the study of Shobal et al., [51] and more recently with Aguilar-Navarro et al., [52]. A much larger proportion of active women appear to be vitamin deficient than active men [53]. This could be the consequence why female athletes use multivitamin-based supplements to a greater extent. Regarding whey protein and creatine, men most consumed these supplements. Previous studies reported that when men were asked about their reasons for using dietary supplements, they revealed that strength and/or muscle mass development was a greater priority for them that it was for women [16]. Meanwhile, whey protein and creatine were the most consumed supplements at all levels of competition. Commonly, supplements with creatine have been used to increase muscle mass and strength during training. However, it has also been reported to improve power and anaerobic capacity [37,54]. Therefore, the use of creatine in racquet sports is of great interest. It has previously been reported that approximately 75% of the top 100 tennis players consumed [18].

Regarding the timing of the ingestion of supplements, this has been shown to play an important role in optimising performance and physical recovery [55]. In the present study, no differences were found regarding when sports supplements were consumed. However, a previous study focussing on fencers did not find differences between levels of competence. Men preferred the intake of sports supplements during sports practice, whereas women supplemented them throughout the day (before, during and after practice, independently) [49].

In the present study, the main reasons for using sports supplements were increased performance and recovery capacity. In fencers, 34.2% reported that the reason for using of sports supplements was an increase in performance. Meanwhile, sailors reported that the purpose of the use of sports supplements was the improvement of performance, there being differences if they were international or national (78% vs. 50%, respectively) [47]. The same was found among handball players [26]. In racquet sports, Lopez-Samanes et al., [18] studying professional tennis players, found that the most important reason for using supplements was to improve performance. Previous studies suggested that, despite having a similar prevalence in dietary supplement use, the reasons for using supplements are slightly different between male and female athletes, as women are usually more concerned about supplements that produce a health benefit [52].

The results obtained in this study show that the most frequent places of purchase of sports supplements were specialised stores and the Internet. The results of the present study coincide with those reported by Wardenaar et al., who found that in a German athlete population, sports supplements were acquired in specialised stores (45%) and on the Internet (25%) [56]. However, they contrast with those reported in fencers where the most frequent places were supermarkets (38.9%) and pharmacies (25%) [49]. Of note is the high amount of supplements bought online, despite the information pointing to a high prevalence of low quality or manipulated products [57]. Although the online market for dietary and sports supplements has facilitated the purchase of these products, some authors believe that this constitutes a public health problem due to the high percentage of supplements available on the Internet with banned or unlabelled substances [57]. In recent years, athletes have been increasing their presence and promotion through social media to reveal their personal lives and develop connections, sponsorships and self-promotion [52].

Regarding whom, if anyone, motivated the players to use supplements in the present study, dieticians/nutritionists and coaches were found to be the most frequent. These results coincide with other studies which analysed the intake of sports supplements in swimmers [25], whose main source of information (40.3%) were coaches. This proved to be the same for gym users [58]. It is widespread for athletes to rely on their dieticians/nutritionists as their main sources of information when selecting the use of a specific dietary supplement [59]. Dietary advice provided by dieticians/nutritionists appears to be associated with a higher prevalence of supplement usage based on scientific evidence [56]. Conversely, athletes who do not receive dietary advice from dieticians/nutritionists appear more likely to consume other supplements with less scientific evidence [56].

Finally, this is the first study focused on the sport of padel that analyses the correlations between supplementation, training and level of play. In this sense, it seems that male padel players increase their intake of supplements as they get older, whereas in the case of female players, apart from age, there is a correlation between the minutes of training and the number of supplements taken as well as a greater perception of the effectiveness of said supplements. Previous studies have suggested that athletes think that the use of supplements is necessary to endure high training loads as well as to improve recovery [60]. In addition, Dascombe et al. [61] concluded that athletes perceived better performance when consuming supplements, also informing that it was important for maintaining of their health. Finally, Heikkinen et al. [62] reported that athletes used supplements to prevent nutritional deficiencies and believed they helped improve their recovery. Recently, another study obtained similar results regarding reasons for supplement intake, with athletes assuring that sports supplements improved their recovery and health [63]. These perceptions could explain the increased intake of supplements in both younger players and older players, both to prevent nutritional deficits and to promote recovery and performance in competition.

The present study may have certain limitations: (i) the sample size was small; (ii) stratification of the data was not taken into consideration in determining the sample size; (iii) the questionnaire that was used to evaluate the consumption of dietary supplements in elite athletes collected information retrospectively. This could lead to inaccurate information on the number and/or type of supplements reported: (iv) participants may have used supplements but did not recognise their use at that time and therefore did not report their use in the questionnaire; (v) a control group was not considered and (vi) the statistical power for the chi-square test was low. Closed questions and a free final option were used to homogenise the answers. Future studies should include more subjects in the sample and use different questionnaires on the topic to support the present findings.

## 5. Conclusions

In conclusion, lower level padel players do not adapt their diet to the physical demands of padel. Moreover, they are not advised by nutrition professionals. Male padel players use more supplements than female padel players, regardless of the level of play. In addition, as the level of the players increases, the number of supplements ingested and the control of the athletes’ diet increases. Meanwhile, creatine in men and vitamin complexes in women were the most used supplements. At a higher level of play, the consumption of creatine supplements, proteins and sports drinks is increased. Finally, although the use of supplements is widespread among athletes, it is important that athletes and the specialists who advise them control their proper and responsible use. Dietary guidelines correctly chosen and planned with different objectives must be based on scientific evidence.

## Figures and Tables

**Table 1:** General characteristics.

	Sex	Low Level	Medium Level	Professional	*p*
*N*	Male	44	42	8	-
	Female	14	11	4	
Age (years)	Male	33.5 ± 11.5	31.9 ± 10.5	29.1 ± 10.3	0.016
	Female	39.6 ± 9.3	38.6 ± 11.2	22.5 ± 5.1	
Weight (kg)	Male **	79.7 ± 11.2	75.5 ± 10.9	74.5 ± 6.7	0.119
	Female	67.8 ± 12.3	62.0 ± 8.4	64.5 ± 4.4	
Height (m)	Male **	1.78 ± 0.05	1.77 ± 0.07	1.79 ± 0.04	0.684
	Female	1.65 ± 0.06	1.65 ± 0.05	1.67 ± 0.05	
BMI	Male	24.5 ± 4.9	21.6 ± 7.4	20.3 ± 8.4	0.194
	Female	23.0 ± 7.5	20.4 ± 7.2	22.9 ± 0.6	
Experience (years)	Male	6.4 ± 6.0	8.3 ± 7.4	15.5 ± 8.7 ++^^	0.064
	Female	8.4 ± 8.1	8.3 ± 7.4	15.5 ± 8.7 ++^^	

BMI: body mass index; *p:* differences between levels; ++ *p* < 0.01 differences mean level vs. professional level; ^^ *p* < 0.01 differences low level vs. professional level; ** *p* < 0.01 differences between sexes.

**Table 2:** Characteristics of the participants’ training and competitions. Values expressed in *n* (%).

Parameters	Sex		Low Level (*n* = 58)	Medium Level (*n* = 53)	Professional (*n* = 12)	*p*	*χ* ^2^
Weekly training sessions (days)	Male (*n* = 94)	1	17 (38.6)	10 (23.8)	0 (0)	<0.001	43.13
		2	16 (36.4)	15 (35.7)	0 (0)		
		≥3	11 (25)	17 (41.4)	8 (100.0)		
	Female (*n* = 29)	1	3 (21.4)	1 (9.1)	0 (0)		
		2	10 (71.4)	6 (54.5)	0 (0)		
		≥3	1 (7.1)	4 (36.4)	4 (100.0)		
	*p*	0.153					
	*χ* ^2^	5.27					
Training duration (min)	Male (*n* = 94)	30–59	4 (9.1)	5 (11.9)	0 (0)	0.014	19.11
		60–89	34 (77.3)	28 (66.7)	4 (50.0)		
		90–119	6 (13.6)	9 (21.4)	2 (25.0)		
		120–179	0 (0)	0 (0)	1 (12.5)		
		180	0 (0)	0 (0)	1 (12.5)		
	Female (*n* = 29)	30–60	3 (21.4)	1 (9.1)	1 (25)		
		60–90	11 (78.6)	8 (72.7)	1 (25)		
		90–120	0 (0)	1 (9.1)	2 (50)		
		120–180	0 (0)	1 (9.1)	0 (0)		
		≥180	0 (0)	0 (0)	(0)		
	*p*	0.550					
	*χ* ^2^	3.05					
Time of training	Male (*n* = 94)	Morning	5 (11.4)	6 (14.3)	3 (37.5)	0.026	17.42
		Afternoon	20 (45.5)	22 (52.4)	1 (12.5)		
		Evening	5 (11.4)	6 (14.3)	1 (12.5)		
		Morning and afternoon	10 (22.7)	6 (14.3)	3 (37.5)		
		Morning and evening	4 (9.1)	2 (4.8)	0 (0)		
	Female (*n* = 29)	Morning	1 (7.1)	2 (18.2)	3 (75)		
		Afternoon	11 (78.6)	7 (63.6)	0 (0)		
		Evening	2 (14.3)	0 (0)	0 (0)		
		Morning and afternoon	0 (0)	2 (18.2)	1 (25)		
		Morning and evening	0 (0)	0 (0)	0 (0)		
	*p*	0.251					
	*χ* ^2^	5.37					
Annual competitions (*n*)	Male (*n* = 94)	0	11 (25)	4 (9.5)	0 (0)	<0.001	62.01
		1–5	29 (65.9)	12 (28.6)	1 (12.5)		
		6–10	3 (6.8)	16 (38.1)	0 (0)		
		>10	1 (2.3)	10 (23.8)	7 (87.5)		
	Female (*n* = 29)	0	0 (0)	0 (0)	0 (0)		
		1–5	11 (78.6)	3 (27.3)	0 (0)		
		6–10	2 (14.3)	4 (36.4)	1 (25)		
		>10	1 (7.1)	4 (36.4)	3 (75)		
	*p*	0.133					
	*χ* ^2^	5.59					

*p* and *χ*^2^ located at the bottom of the sex variables (male and female) indicate the differences between sex. *p* and *χ*^2^ located at the end of the competition level variables (low, medium and professional level) indicate the differences between training levels.

**Table 3:** Dietary characteristics of participants.

Parameters	Sex		Low Level (*n* = 58)	Medium Level (*n* = 53)	Professional (*n* = 12)	*p*	*χ* ^2^
Special diet	Male (*n* = 94)	Yes	11 (25.0)	13 (30.9)	7 (87.5)	0.003	11.59
		No	33 (75.0)	29 (69.1)	1 (12.5)		
	Female (*n* = 29)	Yes	3 (21.4)	6 (54.5)	3 (75.0)		
		No	11 (78.6)	5 (45.5)	1 (25.0)		
	*p*	0.475					
	*χ* ^2^	0.090					
Diet adapted to sports demands	Male (*n* = 94)	Yes	11 (33.0)	13 (31.0)	7 (87.5)	0.001	19.01
		No	33 (75.0)	20 (47.6)	1 (12.5)		
		Don’t know	0 (0)	9 (21.4)	0 (0)		
	Female (*n* = 29)	Yes	3 (21.4)	6 (54.5)	3 (75.0)		
		No	9 (64.3)	5 (45.5)	1 (25.0)		
		Don’t know	2 (14.3)	0 (0)	0 (0)		
	*p*	0.549					
	*χ* ^2^	1.20					
Type of diet	Male (*n* = 94)	Hyperproteic	3 (6.8)	6 (14.3)	2 (25.0)	0.296	14.07
		High in CHO	1 (2.3)	0 (0)	2 (25.0)		
		Low in fat	4 (9.1)	4 (9.5)	0 (0)		
		Mediterranean	2 (4.6)	2 (4.8)	1 (12.5)		
		Ketogenic	1 (2.3)	1 (2.4)	1 (12.5)		
		Hypocaloric	0 (0)	0 (0)	0 (0)		
		Other	0 (0)	0 (0)	1 (12.5)		
	Female (*n* = 29)	Hyperproteic	0 (0)	0 (0)	0 (0)		
		High in CHO	0 (0)	2 (18.2)	2 (50.0)		
		Low in fat	1 (7.5)	2 (18.2)	0 (0)		
		Mediterranean	1 (7.5)	2 (18.2)	1 (25.0)		
		Ketogenic	1 (7.5)	0 (0)	0 (0)		
		Hypocaloric	0 (0)	0 (0)	0 (0)		
		Other	0 (0)	0 (0)	0 (0)		
	*p*	0.078					
	*χ* ^2^	11.36					
Diet control	Male (*n* = 94)	Dietitian/nutritionist	6 (13.6)	6 (14.3)	4 (50.0)	0.366	8.725
		Nobody	1 (2.3)	3 (7.1)	2 (25.0)		
		Personal trainer	2 (4.5)	2 (4.8)	0 (0)		
		Himself/herself	0 (0)	2 (4.8)	1 (12.5)		
		Doctor	2 (4.5)	0 (0)	0 (0)		
	Female (*n* = 29)	Dietitian/nutritionist	2 (14.3)	4 (36.4)	3 (75.0)		
		Nobody	0 (0)	0 (0)	0 (0)		
		Personal trainer	0 (0)	2 (18.2)	0 (0)		
		Himself/herself	0 (0)	0 (0)	0 (0)		
		Doctor	1 (7.1)	0 (0)	0 (0)		
	*p*	0.377					
	*χ* ^2^	4.21					

CHO: carbohydrates; *p* and *χ*^2^ located at the bottom of the sex variables (male and female) indicate the differences between sex. *p* and *χ*^2^ located at the end of the competition level variables (low, medium and professional level) indicate the differences between competition levels. Special diet: a diet that has a special characteristic and limits or does not eat a particular type of food; diet adapted to sporting demands: food intakes based on the competitive period, physical demands of the sport to improve performance and recovery.

**Table 4:** Amount, type and timing of sports supplements in study participants.

Parameters	Sex		Low Level (*n* = 58)	Medium Level (*n* = 53)	Professional (*n* = 12)	*p*	*χ* ^2^
Sports supplements	Male (*n* = 94)	Yes	22 (50.0)	21 (50.0)	7 (87.5)	0.010	9.11
		No	22 (50.0)	21 (50.0)	1 (12.5)		
	Female (*n* = 29)	Yes	4 (28.6)	4 (36.4)	4 (100)		
		No	10 (71.4)	7 (63.6)	0 (0)		
	*p*	0.184					
	*χ* ^2^	1.23					
Amount (*n*)	Male (*n* = 94)	1	5 (11.4)	3 (7.1)	0 (0)	0.463	7.70
		2	7 (15.9)	8 (19.0)	1 (12.5)		
		3	3 (6.8)	6 (14.3)	3 (37.5)		
		4	4 (9.1)	1 (2.4)	1 (12.5)		
		≥5	3 (6.8)	4 (9.5)	2 (25.0)		
	Female (*n* = 29)	1	3 (21.4)	3 (27.3)	1 (25.0)		
		2	1 (7.1)	1 (9.1)	1 (25.0)		
		3	0 (0)	0 (0)	1 (25.0)		
		4	0 (0)	0 (0)	0 (0)		
		≥5	0 (0)	0 (0)	1 (25.0)		
	*p*	0.032					
	*χ* ^2^	10.58					
Days of consumption	Male (*n* = 94)	Training sessions	6 (13.6)	4 (9.5)	0 (0)	0.016	18.71
		Competition	1 (2.3)	1 (2.4)	0 (0)		
		Training sessions and competition	5 (11.4)	3 (7.1)	2 (25.0)		
		Daily	9 (20.5)	12 (28.6)	1 (12.5)		
		In all the above cases	1 (2.3)	2 (4.8)	4 (50.0)		
	Female (*n* = 29)	Training sessions	1 (7.1)	1 (9.1)	0 (0)		
		Competition	0 (0)	0 (0)	0 (0)		
		Training sessions and competition	0 (0)	1 (9.1)	2 (50.0)		
		Daily	3 (21.4)	2 (18.2)	1 (25.0)		
		In all the above cases	0 (0)	0 (0)	1 (25.0)		
	*p*	0.913					
	*χ* ^2^	0.977					
Time of consumption	Male (*n* = 94)	Before training/ competition	4 (9.1)	8 (19.0)	0 (0)	0.219	8.26
		During training/ competition	2 (4.5)	1 (2.4)	0 (0)		
		After training/ competition	9 (20.5)	7 (16.7)	4 (50.0)		
		In all the above cases	7 (15.9)	6 (14.3)	3 (37.5)		
	Female (*n* = 29)	Before training/ competition	3 (21.4)	3 (27.3)	0 (0)		
		During training/ competition	0 (0)	0 (0)	1 (25.0)		
		After training/ competition	1 (7.1)	1 (9.1)	3 (75.0)		
		In all the above cases	0 (0)	0 (0)	0 (0)		
	*p*	0.259					
	*χ* ^2^	4.020					

*p* and *χ*^2^ located at the bottom of the sex variables (male and female) indicate the differences between sex. *p* and *χ*^2^ located at the end of the competition level variables (low, medium and professional level) indicate the differences between competition levels. Sport supplements: food, food component, or nutrient that is purposefully ingested, in addition to the habitually consumed diet, with the aim of achieving a specific physical performance or health benefit.

**Table 5:** Acquisition, motivation and control of sports supplements.

Parameters	Sex		Low Level (*n* = 58)	Medium Level (*n* = 53)	Professional (*n* = 12)	*p*	*χ*²
Reason for supplements	Male (*n* = 94)	Increase performance	7 (15.9)	10 (23.8)	4 (50.0)	0.212	15.57
		Improve physical appearance	1 (2.3)	1 (2.4)	0 (0)		
		Take care of health	3 (6.8)	6 (14.3)	1 (12.5)		
		Improve recovery	9 (20.5)	3 (7.1)	2 (25.0)		
		For health problems	1 (2.3)	0 (0)	0 (0)		
		Reduce deficit	0 (0)	2 (4.8)	0 (0)		
		Other	1 (2.3)	0 (0)	0 (0)		
	Female (*n* = 29)	Increase performance	1 (7.1)	1 (9.1)	3 (75.0)		
		Improve physical appearance	0 (0)	2 (18.2)	1 (25.0)		
		Take care of health	2 (14.3)	0 (0)	0 (0)		
		Improve recovery	0 (0)	0 (0)	0 (0)		
		For health problems	1 (7.1)	0 (0)	0 (0)		
		Reduce deficit	0 (0)	1 (9.1)	0 (0)		
		Other	0 (0)	0 (0)	0 (0)		
	*p*	0.100					
	*χ*²	10.65					
Where do you get your supplements?	Male (*n* = 94)	Specialty store	6 (13.6)	8 (19.0)	1 (12.5)	0.364	13.06
		Internet	13 (29.5)	8 (19.9)	4 (50.0)		
		Dietitian/nutritionist	1 (2.3)	1 (2.4)	1 (12.5)		
		Pharmacy	1 (2.3)	2 (4.8)	0 (0)		
		Other	1 (2.3)	1 (2.4)	1 (12.5)		
	Female (*n* = 29)	Specialty store	2 (14.3)	2 (18.2)	0 (0)		
		Internet	1 (7.1)	0 (0)	3 (75.0)		
		Dietitian/nutritionist	0 (0)	1 (9.1)	0 (0)		
		Pharmacy	1 (7.1)	0 (0)	0 (0)		
		Other	0 (0)	1 (9.1)	1 (25.0)		
	*p*	0.852					
	*χ*²	2.647					
Who motivated you to consume them?	Male (*n* = 94)	Internet	3 (6.8)	4 (9.5)	0 (0)	0.171	16.45
		Coach	5 (11.4)	3 (7.1)	3 (37.5)		
		Dietitian/nutritionist	9 (20.5)	3 (7.1)	3 (37.5)		
		Friend	4 (9.1)	4 (9.5)	1 (12.5)		
		Doctor	0 (0)	3 (7.1)	0 (0)		
		Advertising	0 (0)	0 (0)	0 (0)		
		Myself	1 (2.3)	5 (11.9)	0 (0)		
	Female (*n* = 29)	Internet	2 (14.3)	0 (0)	0 (0)		
		Coach	0 (0)	0 (0)	0 (0)		
		Dietitian/nutritionist	0 (0)	2 (18.2)	4 (100.0)		
		Friend	1 (7.1)	0	0 (0)		
		Doctor	1 (7.1)	0	0 (0)		
		Advertising	0 (0)	1 (9.1)	0 (0)		
		Myself	0 (0)	1 (9.1)	0 (0)		
	*p*	0.206					
	*χ*²	8.467					

**Table 6:** Perception of the effectiveness of supplements in the performance and quantity of supplements.

Parameters	Sex	Low Level (*n* = 58)	Medium Level (*n* = 53)	Professional (*n* = 12)	*p*
Perception (pto)	Male (*n* = 94)	7.68 ± 1.42	7.33 ± 2.28	8.00 ± 1.29	0.337
	Female (*n* = 29)	7.50 ± 1.00	7.25 ± 1.89	8.75 ± 1.50	
Number of sports supplements (*n*)	Male (*n* = 94)	2.68 ± 1.39	3.41 ± 2.98	4.14 ± 2.34	0.786
	Female * (*n* = 29)	1.25 ± 0.50	1.35 ± 0.45	3.25 ± 2.63	

* *p* < 0.05 male vs. female.

**Table 7:** Correlations according to sex in the different variables analysed.

Number of Supplements				
	Male (*n* = 94)		Female (*n* = 29)	
	*r*	*p*	*r*	*p*
Weekly training sessions (*n*)	0.108	0.451	0.548	0.085
Training sessions (min)	0.047	0.697	0.697	0.012
Competition level	0.219	0.123	0.493	0.103
**Supplement intake**				
	Male (*n* = 94)		Female (*n* = 29)	
	*r*	*p*	*r*	*p*
Age (years)	0.530	<0.001	0.412	0.029
Experience (years)	0.045	0.763	0.309	0.456
**Perception of the effectiveness of sports supplements**				
	Male (*n* = 94)		Female (*n* = 29)	
	*r*	*p*	*r*	*p*
Weekly training sessions (*n*)	−0.076	0.599	0.510	0.091
Training sessions (min)	0.206	0.152	0.592	0.042
Competition level	0.011	0.939	0.349	0.266

*r*: Pearson correlation coefficient.

## Data Availability

Not applicable.
